# Supplementary figures and images for: Addition of Tocilizumab to the Standard of Care Reduces Mortality in Severe COVID-19: A Systematic Review and Meta-Analysis
Source: Front Med (Lausanne). 2020 Oct 2;7:586221. doi: 10.3389/fmed.2020.586221 (PMC7566918; doi:10.3389/fmed.2020.586221)

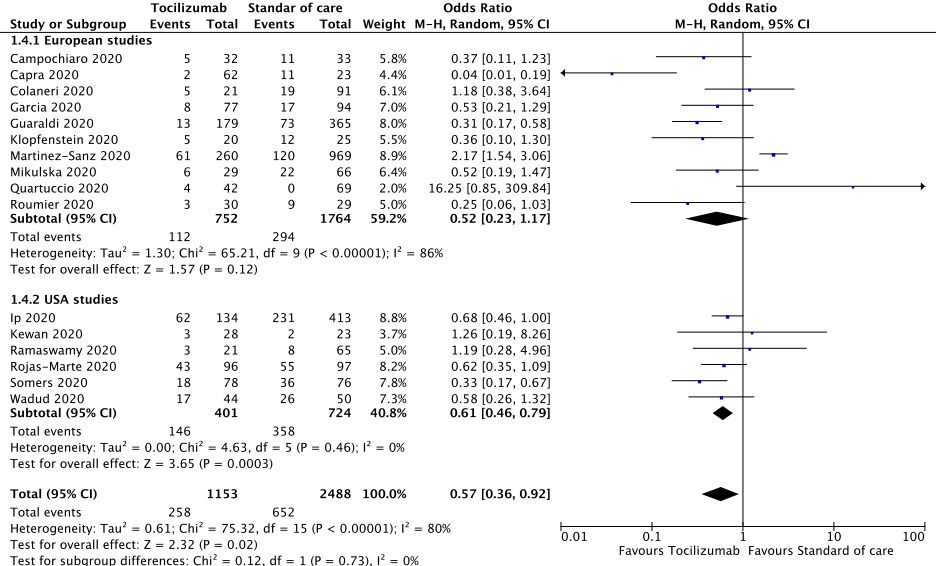

Supplement: Supplementary Figure 1 — Forest plot for sensitivity analysis for the American and European studies. [file Image_1.JPEG]

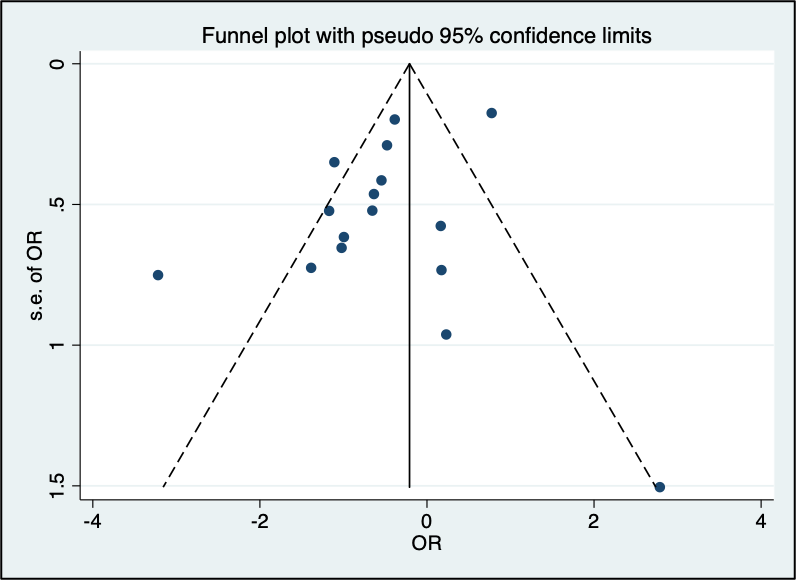

Supplement: Supplementary Figure 2 — Funnel plot analysis of publication bias. [file Image_2.JPEG]
