# Supplementary material for: Addition of Tocilizumab to the Standard of Care Reduces Mortality in Severe COVID-19: A Systematic Review and Meta-Analysis
Source: Front Med (Lausanne). 2020 Oct 2;7:586221. doi: 10.3389/fmed.2020.586221 (PMC7566918; doi:10.3389/fmed.2020.586221)
Supplement: Supplementary Table 1 — Risk of Bias Assessment Tool for Non-randomized Studies (RoBANS) summary of risk of bias assessment. [file Table_1.docx]

**Supplementary Table 1:** Risk of Bias Assessment Tool for Non-randomized Studies

(RoBANS) summary of risk of bias assessment

| Study | Selection | | Performance | Detection | Attrition | Reporting |
| --- | --- | --- | --- | --- | --- | --- |
|  | Selection of Participants | Confounding variables | Measurement of exposure | Blinding of outcome assessment | Incomplete outcome data | Selective outcome reporting |
| Campochiaro et al. | Unclear | Low | Low | Unclear | Low | Low |
| Capra et al. | Unclear | Low | Low | Unclear | Low | Low |
| Colaneri et al. | Unclear | High | Low | Unclear | Low | Low |
| Klopfenstein et al. | Unclear | Low | Low | Unclear | Low | Low |
| Quartuccio et al. | Unclear | High | Low | Unclear | Low | Low |
| Roumier et al. | Unclear | High | Low | Unclear | Low | Unclear |
| Wadud et al. | Unclear | High | Low | Unclear | Low | Low |
| Ramaswamy et al. | Unclear | High | Low | Unclear | Low | Low |
| Ip et al. | Unclear | High | Low | Unclear | Low | Low |
| Somers et al. | Unclear | High | Low | Unclear | Low | Low |
| Kewan et al. | Unclear | High | Low | Unclear | Low | Low |
| Martinez-Sanz et al. | Unclear | High | Low | Unclear | Low | Low |
| Guaraldi et al. | Unclear | Low | Low | Unclear | Low | Low |
| Mikulska et al. | Unclear | High | Low | Unclear | Low | Low |
| Garcia et al. | Unclear | High | Low | Unclear | Low | Low |
| Rojas-Marte et al. | Unclear | High | Low | Unclear | Low | Low |
